# Efficacy and Safety of Herbal Supplements with Anxiolytic, Antidepressant, and Sedative Action: A Review of Clinical Data and Toxicological Risks

**DOI:** 10.3390/ph19030399

**Published:** 2026-02-28

**Authors:** Maria-Nina Căuș, Mariana Lupoae, Carmen Lidia Chițescu

**Affiliations:** Research Centre in the Medical-Pharmaceutical Field, Faculty of Medicine and Pharmacy, “Dunarea de Jos” University, 800201 Galati, Romaniamariana.lupoae@ugal.ro (M.L.)

**Keywords:** adulteration, pharmacologic interactions, regulatory gaps, toxic effect

## Abstract

**Background/Objectives:** Plant-based supplements are widely used for the management of anxiety, depression, and insomnia. Despite their over-the-counter availability and perceived safety, these products may pose relevant pharmacological and toxicological risks. This narrative review critically evaluates clinical evidence on commonly used herbal preparations, with particular emphasis on herb–drug interactions, adverse effects, and issues related to product adulteration. **Methods:** Major scientific databases (PubMed, Scopus, and Web of Science) were searched to identify clinical studies evaluating plant-based supplements for mental health and sleep disorders. Data on study design, dosage, efficacy, and adverse events were analyzed, together with regulatory information and reports of product adulteration and quality concerns. **Results:** Herbal supplements such as *Hypericum perforatum*, *Passiflora incarnata*, *Valeriana officinalis*, *Piper methysticum*, *Withania somnifera*, *Crocus sativus*, and *Curcuma longa* demonstrated anxiolytic, antidepressant, and sedative effects in clinical studies, with improvements in mood, stress levels, and sleep quality. Proposed mechanisms include modulation of monoaminergic and GABAergic pathways, serotonergic activity, regulation of the hypothalamic–pituitary–adrenal axis, and anti-inflammatory effects. However, clinically relevant risks were identified, including cytochrome P450–mediated drug interactions, excessive sedation, serotonin syndrome, and toxic effects associated with adulterated products, such as hepatotoxicity, cardiovascular events, and neurological disturbances. **Conclusions:** While plant-based supplements may provide clinically meaningful benefits for anxiety, depression, and insomnia, their use requires careful clinical monitoring due to potential pharmacokinetic and pharmacodynamic interactions and safety concerns. Increased awareness of herb–drug interactions and stricter quality control are essential to optimize therapeutic outcomes and minimize harm.

## 1. Introduction

Supplements based on plant extracts are frequently used to treat anxiety, depression symptoms and sleep disorders. Although these products are available without a prescription and are generally thought to be safe, there are pharmacological and toxicological risks associated with their use [1].

Over the past 20 years, there has been a significant increase in the use of dietary supplements. The global dietary supplements market was valued at USD 152 billion in 2021. According to the latest STATISTA report, the global market is expected to reach USD 300 billion by 2028 [2]. In 2021, the global market size for neuroprotective supplements was estimated at USD 7.6 billion, with growth expected to reach USD 15.59 billion by 2030 [3]. Most herbal medicines used in the phytopharmacology of depression are non-prescription products or dietary supplements and are considered safe, causing fewer adverse reactions compared to conventional pharmaceuticals [4].

Dietary supplements that are frequently used and have a high market value are currently more susceptible to economic fraud, which can take the form of adding out-of-label components, incorrect amounts, unauthorized components, or using lower-quality raw materials [5].

Within the European Union, the regulation of dietary supplements and pharmaceutical drugs is governed by two distinct legislative frameworks, which reflect fundamental differences in terms of purpose, the nature of the products, and the requirements for safety and efficacy evaluation and consumer information [6,7,8,9,10]. These regulatory contrasts are summarized in Table 1, which provides a comparative overview of the main European rules applicable to food supplements and medicines.

In summary, the regulatory distinction between dietary supplements and medicinal products in the European Union reflects substantial differences in pre-market evaluation, quality control, efficacy demonstration, and post-marketing surveillance [6,7,8]. While medicines involve documented and controlled intervention, supplements largely remain within the realm of self-regulation and individual responsibility. The differences in the regulatory framework allow for broad consumer access to plant-based products; it also creates potential gaps in safety monitoring and risk communication. In the absence of adverse-reaction-reporting mechanisms comparable to the pharmacovigilance system applied to medicines, the potential risks of supplements may remain underreported or insufficiently recognized [12].

Moreover, laboratory tests of such products often reveal that food supplements contain various undeclared chemical substances [13], which can trigger a range of serious adverse reactions, complications of pre-existing conditions, and drug interactions [14]. Without strict regulations, there is a risk that food supplements may interact with prescribed medications, cause unexpected side effects, or be contaminated with harmful substances, including heavy metals, pesticides, and pathogenic microorganisms [15].

Recognizing these regulatory and safety gaps is essential for interpreting the clinical evidence and toxicological risks discussed throughout this review.

## 2. Results and Discussions

The reviewed plant-based supplements, including *Hypericum perforatum*, *Passiflora incarnata*, *Valeriana officinalis*, *Piper methysticum*, *Withania somnifera*, *Crocus sativus*, and *Curcuma longa*, demonstrate clinically relevant anxiolytic, antidepressant, sedative, and neuroprotective effects [16]. Their mechanisms—modulation of monoamine neurotransmitters, GABAergic activity, serotonin receptor sensitivity, and regulation of the HPA (hypothalamic-pituitary-adrenal) axis and inflammatory pathways—support their potential role in managing anxiety, depression, and sleep disorders. These findings are largely consistent with previous clinical studies and meta-analyses, confirming improvements in mood, stress reduction, and sleep quality [16].

However, the pharmacological and toxicological risks associated with these supplements must be considered. Evidence from the U.S. Food and Drug Administration (FDA) and the Rapid Alert System for Food and Feed (RASFF) databases highlights frequent adulteration of supplements with undeclared pharmacologically active compounds, including benzodiazepines, antidepressants, central nervous system (CNS) stimulants, and cholinergic agents. Such adulteration increases the risk of drug interactions, serotonin syndrome, excessive sedation, and other adverse events. Regulatory discrepancies between dietary supplements and pharmaceutical drugs, particularly regarding pre-market efficacy and safety evaluation, further complicate their safe use [17].

### 2.1. Clinical and Epidemiological Aspects of Anxiety, Depression, and Sleep Disorders

Anxiety is a feeling of apprehension typically manifested as an exaggerated emotional response to situations perceived, often subconsciously, as threatening. It is generally accompanied by muscle rigidity, restlessness, fatigue, and impaired concentration. Chronic anxiety induces neurochemical alterations in the brain and elevates levels of stress hormones, which subsequently exacerbate symptoms such as dizziness, headaches, and depression in both frequency and severity [18]. The prevalence and comorbidity of anxiety disorders differ significantly between women and men, with a greater burden reported among women [18]. Pharmacological management of anxiety involves the following medications with associated adverse effects. Anxiety is commonly treated with benzodiazepines, SSRIs, and SNRIs, which may cause sedation, cognitive impairment, tolerance, and dependence [19].

Depression is a major mood disorder characterized by a persistent low mood, profound sadness, cognitive dysfunction, and loss of interest or pleasure [20,21]. Clinical features include feelings of hopelessness, emptiness, and despair. The disorder may also present with anhedonia, psychomotor disturbances, alterations in sleep and appetite, impaired concentration, and suicidal ideation [21]. Clinically diagnosed depression is most common among middle-aged adults, whereas mood disturbances, such as low mood, fatigue, or loss of interest, are particularly prevalent in young women [22].

Approximately 280 million people worldwide live with depression, according to the World Health Organization (WHO) [23]. The COVID-19 pandemic has led to a significant increase in the incidence of depression, with an estimated global rise of 28.1%, notably impacting older adults [24].

The treatment of depression involves both pharmacological and non-pharmacological approaches, including, in particular, psychotherapeutic techniques [25]. Several classes of antidepressants are used in the pharmacotherapy of this disorder, including selective serotonin reuptake inhibitors (SSRIs), tricyclic antidepressants (TCAs), serotonin-norepinephrine reuptake inhibitors (SNRIs), norepinephrine reuptake inhibitors (NRIs), and norepinephrine-dopamine reuptake inhibitors (NDRIs), monoamine oxidase inhibitors (MAOIs, both A and B), serotonin modulators, atypical antidepressants, and other agents acting on central neurotransmitter systems [26].

These medications are associated with a range of adverse effects: TCAs can cause toxic delirium, grand mal convulsions, increased liver enzymes, urinary retention, facial flushing, and cardiovascular complications such as orthostatic hypotension; SSRIs commonly produce psychological and neurological effects, gastrointestinal disturbances, sexual dysfunction, hyponatremia, and, rarely, serotonin syndrome; SNRIs and NRIs may cause headache, nausea, and hypertension; and MAOIs can provoke nausea, vomiting, and dietary interactions [26,27,28].

The prevalence of sleep disorders may vary according to the patient’s associated conditions. Studies indicate that diabetes is associated with sleep-disordered breathing, short sleep duration, and non-restorative sleep [29]. Sleep disorders are common across all age groups and often co-occur with anxiety and depression. Clinical presentation differs by age: adults typically report excessive daytime sleepiness, whereas children more often exhibit motor hyperactivity, inattention, irritability, or oppositional behavior [30]. Cognitive–behavioral therapy for insomnia (CBT-I) is the first-line non-pharmacological treatment, and it incorporates behavioral strategies such as sleep restriction, stimulus control, relaxation techniques, and sleep hygiene education [31,32].

Untreated sleep disorders increase the risk of accidents, mood and anxiety disorders, false memory, and cognitive decline [33,34]. In addition, they are associated with a higher risk of cerebrovascular and cardiovascular disease, particularly in individuals with severe obstructive sleep apnea and excessive daytime sleepiness [35].

Sleep disorders are managed with benzodiazepines, non-benzodiazepine hypnotics (Z-drugs), melatonin receptor agonists, or orexin receptor antagonists, which may lead to residual daytime sedation, tolerance, dependence, or complex sleep-related behaviors [36].

### 2.2. Mechanisms of Action of Natural Compounds in Anxiety, Depression, and Sleep Disorders

Medicinal plants are widely used in many countries to treat CNS disorders, and there is a growing interest in exploring natural products with activity on the CNS [37,38].

Plants such as St. John’s Wort (*Hypericum perforatum*), Passionflower (*Passiflora incarnata*), Valerian (*Valeriana officinalis*), Kava (*Piper methysticum*), Ashwagandha (*Withania somnifera*), Saffron (*Crocus sativus*), and Turmeric (*Curcuma longa*), among others, have been rigorously tested in clinical studies conducted over the past 10–15 years [37,38,39]. Certain plant-based antidepressant supplements offer promising prospects in the treatment of these disorders through well-established psychopharmacological mechanisms [39]. These include inhibition of monoamine reuptake (serotonin, dopamine, and norepinephrine), enhanced binding and increased sensitivity of serotonin receptors, monoamine oxidase inhibition [39,40,41]. Other effects may include GABAergic activity, cytokine modulation (particularly in depressive disorders associated with comorbid inflammatory conditions), and influences on the opioid and endocannabinoid systems [40].

The primary mechanisms of action are based on the modulation of neuronal communication, which is largely mediated by secondary metabolites such as alkaloids, flavonoids, terpenoids, phenolic acids and other bioactive constituents [37,38,39]. These compounds interact with neurotransmitter and neuromodulator receptors, influence neurotransmitter synthesis, and modulate overall CNS function [42]. In addition to these mechanisms, phytochemicals may exert further effects through modulation of neuronal communication pathways at both synaptic and intracellular levels. Additional mechanisms include interactions with ion channels, modulation of second-messenger systems, and influences on neuroendocrine signaling involved in stress and mood regulation [43]. Through these complementary pathways, these compounds contribute to a broad spectrum of psychotropic effects, including antidepressant, anxiolytic, cognitive-enhancing (nootropic), sedative, hypnotic, and analgesic activities [38,39].

#### 2.2.1. Mechanisms of Action of *Hypericum perforatum*

Regarding the antidepressant effect of St. John’s Wort, the flowering aerial parts of the plant are commonly used for treatment, and this activity has been primarily attributed to hyperforin [44,45]. The standardized extracts used in clinical studies generally contain approximately 0.3% hypericin and 2–5% hyperforin [46]. Although hypericin was initially believed to be responsible for the antidepressant effects, subsequent research has demonstrated that hyperforin plays a key role in this activity [43,47].

Hyperforin exerts an inhibitory effect on the reuptake of several key neurotransmitters involved in mood regulation, including serotonin, norepinephrine, and dopamine, contributing to its antidepressant properties [45,46,48]. Additionally, in vitro studies have shown that hyperforin, at concentrations of 0.1–1 μM, produces non-specific presynaptic effects, leading to the inhibition of uptake for a wide range of neurotransmitters, including choline [48] gamma-aminobutyric acid (GABA) [49], and glutamate [49,50]. Moreover, studies have demonstrated that hyperforin inhibits serotonin reuptake in a dose-dependent manner, further supporting its significant role in the antidepressant mechanism of St. John’s Wort [47]. This inhibition is not based on specific binding sites on transporter molecules; rather, the mechanism of action appears to be associated with sodium ion transport pathways, leading to an increase in intracellular Na^+^ concentration [47].

#### 2.2.2. Mechanisms of Action of *Passiflora incarnata*

The aerial parts of the plant, flowers, and fruits are used for medicinal purposes. The main phytochemical compounds found in *Passiflora incarnata* are flavonoids (apigenin, luteolin, quercetin, and kaempferol) and flavonoid glycosides (vitexin, isovitexin, orientin, and isoorientin), with isovitexin being the most abundant [51,52]. Numerous pharmacological effects of *Passiflora incarnata* are mediated through modulation of the GABAergic system, including affinity for GABA_A_ and GABA_B_ receptors, as well as influencing GABA uptake [53,54,55].

#### 2.2.3. Mechanisms of Action of *Valeriana officinalis*

*Valeriana officinalis* contains active constituents such as iridoids, flavonoids, and essential oils, including monoterpenes and sesquiterpenes [56,57]. Its anxiolytic and sedative effects are primarily attributed to the stimulation of GABAergic activity via valerenic acid [58,59]. Additionally, valerian may modulate neurotransmitters such as serotonin and noradrenaline, which explains its role in stress reduction and sleep improvement [59].

#### 2.2.4. Mechanisms of Action of *Piper methysticum*

The principal bioactive constituents of *Piper methysticum* (kava) are kavalactones, including kavain, dihydrokavain, methysticin, dihydromethysticin, yangonin, desmethoxyyangonin [60]. Multiple studies have demonstrated that various kava extracts and isolated kavalactone compounds exert modulatory effects on the GABAergic system, inhibit MAO-B activity, and interact with 5-HT1A serotonin receptors [61]. In vivo pharmacological evaluations in rodent models have revealed sedative, anxiolytic, and muscle-relaxant properties of these extracts and isolated molecules [62]. Furthermore, emerging evidence indicates additional pharmacodynamic actions, including anticonvulsant, spasmolytic, neuroprotective, and analgesic effects [63].

#### 2.2.5. Mechanisms of Action of *Crocus sativus*

The stigmas of saffron (*Crocus sativus*) contain approximately 40–50 bioactive therapeutic compounds, including around 30% crocin, 5–15% picrocrocin, and over 5% volatile constituents such as safranal [64,65]. The antidepressant activity of saffron is primarily attributed to crocin’s inhibition of dopamine and norepinephrine reuptake, and safranal’s inhibition of serotonin reuptake [66]. Safranal, a monoterpenoid aldehyde derived from saffron essential oil, exhibits multiple other biological activities including antihyperglycemic, anti-inflammatory, antioxidant, anticonvulsant, and anxiolytic effects [64].

#### 2.2.6. Mechanisms of Action of *Curcuma longa*

*Curcuma longa*, known for its main active compound curcumin [67], exhibits multiple anti-inflammatory, antioxidant, neuroprotective, and immunomodulatory properties [67,68,69]. Several studies have demonstrated curcumin’s ability to modulate neurotransmitter levels, inflammatory pathways, excitotoxicity, neuroplasticity, hypothalamic–pituitary–adrenal axis dysfunction, insulin resistance, oxidative and nitrosative stress, and the endocannabinoid system [68,69,70]. These diverse mechanisms of action attributed to curcumin may influence key pathophysiological processes involved in the development of major depressive disorder [69].

#### 2.2.7. Mechanisms of Action of *Withania somnifera*

*Withania somnifera*, commonly known as Ashwagandha, contains withanolides as its main active compounds, which are responsible for most of the plant’s therapeutic effects, including anti-inflammatory, antioxidant, adaptogenic, anxiolytic, and antidepressant properties [71]. The results from multiple studies, including randomized controlled trials and systematic reviews, suggest that Ashwagandha positively influences these health aspects through its action on the HPA axis, modulation of neurotransmitters (GABA, serotonin), and antioxidant activity [71,72]. Additionally, Ashwagandha has been shown to significantly reduce stress and modulate cortisol levels, indicating an effective adaptogenic effect [73].

Table 2 summarizes selected medicinal plants with anxiolytic, antidepressant, and sedative properties, highlighting their main mechanisms of action and the adverse reactions associated with their use.

### 2.3. Clinical Studies on the Anxiolytic, Antidepressant, and Sedative Effects of Medicinal Plants

There has been a significant increase in interest regarding the use of medicinal plants for the treatment of neuropsychiatric disorders such as anxiety, depression, and sleep disturbances [61]. Numerous clinical studies have investigated the efficacy and mechanisms of action of plant extracts with anxiolytic, antidepressant, and sedative properties, highlighting their potential as alternative or complementary therapeutic options [74,75,76,77,78,79].

Examples of clinical studies investigating the effects of well-known medicinal plants on anxiety symptoms, depression, and sleep disorders are presented below.

#### 2.3.1. *Hypericum perforatum*

In a randomized, double-blind study carried out over 12 weeks on 135 patients with major depressive disorder, a standardized *Hypericum perforatum* extract (900 mg/day) was compared against fluoxetine (20 mg/day) and placebo. The findings revealed that *Hypericum perforatum* was significantly more effective than fluoxetine and showed a tendency toward superiority over placebo [75]. Furthermore, a comparative analysis [80] demonstrated that *Hypericum perforatum* had better tolerability compared to paroxetine, with the synthetic drug exhibiting a 10- to 38-times higher rate of adverse events.

The primary objective of a multicenter, double-blind study was to demonstrate the non-inferiority of the *Hypericum perforatum* extract STW3-VI (900 mg) compared to the SSRI citalopram (20 mg), as well as the superiority of *Hypericum perforatum* over placebo. The study involved 388 outpatients diagnosed with moderate depression. Safety and tolerability of the *Hypericum perforatum* extract versus citalopram and placebo were assessed using the Clinical Global Impression (CGI) scale, the incidence of adverse events, laboratory parameters, and vital sign monitoring. Based on these data, a statistically significant therapeutic equivalence between *Hypericum perforatum* extract STW3-VI and citalopram was established, along with the extract’s superiority over placebo [78].

#### 2.3.2. *Passiflora incarnata*

In a double-blind, placebo-controlled trial, 128 patients were randomized into two groups: one group (*n* = 68) received oral *Passiflora incarnata*, while the other group (*n* = 60) was administered 10 mg of oxazepam as premedication 90 min prior to surgery. Anxiety levels were measured using a Numeric Rating Scale (NRS) both before and 90 min after premedication. The results demonstrated that oral *Passiflora incarnata* effectively reduced preoperative anxiety in outpatient surgery, with efficacy comparable to that of oral oxazepam [51].

A total of 65 opioid-dependent patients were randomly assigned to receive either *Passiflora incarnata* extract combined with clonidine tablets or clonidine tablets plus placebo drops during a 14-day double-blind clinical trial. The fixed daily dose consisted of 60 drops of *Passiflora incarnata* extract and a maximum daily dose of 0.8 mg clonidine administered in three divided doses. The severity of opioid withdrawal syndrome was assessed on days 0, 1, 2, 3, 4, 7, and 14 using the Short Opioid Withdrawal Scale (SOWS). Both treatment protocols were equally effective in alleviating the physical symptoms of withdrawal. However, the group of *Passiflora incarnata* plus clonidine demonstrated significant superiority over clonidine alone in managing psychological symptoms. These findings suggest that *Passiflora incarnata* extract may serve as an effective adjunctive agent in the management of opioid withdrawal [55].

Another study was conducted on 36 patients diagnosed with generalized anxiety disorder (GAD) according to the Diagnostic and Statistical Manual of Mental Disorders (DSM)-IV criteria. According to DSM-IV, GAD is characterized by excessive anxiety and worries occurring on most days for at least six months, regarding a variety of events or activities, and is difficult to control. Patients were randomly assigned to two groups: 18 received *Passiflora incarnata* extract (45 drops/day) plus placebo tablets, and 18 received oxazepam (30 mg/day) plus placebo drops, over a 4-week period. Both *Passiflora incarnata* extract and oxazepam proved effective in treating generalized anxiety disorder, with no significant difference observed between the two treatment groups at the study’s conclusion. Oxazepam demonstrated a faster onset of action; however, significantly more work-performance impairment was reported among subjects treated with oxazepam [54].

*Passiflora incarnata* extracts have demonstrated anxiolytic and sedative properties in several clinical studies [81]. Placebo-controlled trials reported significant reductions in anxiety symptoms, while comparative studies indicated effects comparable to those of benzodiazepines [55]. In addition to its anxiolytic action, sedative properties of Passiflora extracts have also been confirmed in clinical investigations [55,81]. A randomized, double-blind study involving 36 patients with generalized anxiety disorder compared *Passiflora incarnata* extract with oxazepam and found no significant differences in overall anxiolytic efficacy between the two treatments [55]. Although oxazepam was associated with a more rapid onset of symptom relief, participants receiving *Passiflora* reported greater impairment in work performance, suggesting differences in tolerability and functional outcomes between the two interventions [54,55].

#### 2.3.3. *Valeriana officinalis*

A total of 202 outpatients diagnosed with non-organic insomnia were treated in a randomized, double-blind study comparing 600 mg/day of valerian extract (LI 156) with 10 mg/day of oxazepam over a period of 6 weeks. Sleep quality (SQ) after 6 weeks, assessed using the Sleep Questionnaire B, demonstrated that valerian extract at 600 mg/day (LI 156) was at least as effective as oxazepam 10 mg/day. Both treatments significantly improved sleep quality compared to baseline values [82].

In a double-blind clinical trial, 60 patients aged between 15 and 60 years were randomly assigned to two groups of 30 each. At 9 PM, prior to surgery, Group 1 received 10 drops of valerian oil, while Group 2 received 5 mg of diazepam mixed in 50 mL of water. The Spielberger Anxiety Questionnaire was used to assess anxiety levels before the intervention and one hour prior to the start of surgery. Comparison between the two groups revealed no significant differences, with valerian proving to be as effective as diazepam but with fewer side effects [83].

#### 2.3.4. *Withania somnifera*

A non-blinded, randomized study involved 80 patients diagnosed with major depressive disorder. Patients were randomly divided into two groups: one group received routine treatment with sertraline (Sertraline Group), and the other group received ashwagandha 250 mg twice daily along with sertraline (Sertraline + Ashwagandha Group). All liver function parameters were assessed before treatment initiation and reassessed after 3 months of medication in both groups. In the sertraline group, levels of AST (aspartate aminotransferase), ALT (alanine aminotransferase), total proteins, and globulins significantly increased, whereas treatment with sertraline plus ashwagandha had no effect on liver parameters [84].

A randomized, double-blind, placebo-controlled clinical trial evaluated the effects of a sustained-release Ashwagandha root extract capsule (300 mg, Prolanza™; hereafter referred to as Ashwagandha SR) on cognitive function, stress levels, sleep quality, overall well-being, and safety in stressed subjects. Compared to the placebo group, treatment with one Ashwagandha SR capsule once daily for 90 days significantly improved memory and concentration, psychological well-being, and sleep quality, reduced stress levels, and was safe and well tolerated. No adverse events were reported [85].

#### 2.3.5. Kava Kava

A randomized, double-blind, multicenter clinical trial conducted over 8 weeks investigated the effect of Kava-Kava *LI 150*, a standardized kava extract, in patients with generalized anxiety disorder, involving 129 participants, compared to buspirone (10 mg/day) or opipramol (100 mg/day). Approximately 75% of patients in each treatment group were classified as responders, defined by a 50% reduction in the Hamilton Anxiety Rating Scale (HAMA) score, and around 60% achieved full remission [86].

In a double-blind study, the efficacy and safety of a standardized kava extract were evaluated for the treatment of generalized anxiety disorder in 37 patients over a 4-week period, compared to placebo. Improvements were observed in both treatment groups, but no significant differences were found compared to placebo. Both treatments were well tolerated [87].

#### 2.3.6. *Crocus sativus*

In a randomized, double-blind, placebo-controlled trial, 123 participants diagnosed with major depressive disorder were assigned to one of four treatment groups: placebo, low-dose curcumin extract (250 mg twice daily), high-dose curcumin extract (500 mg twice daily), or a combination of low-dose curcumin extract plus saffron (15 mg twice daily) for 12 weeks. Compared to placebo, treatments with curcumin and saffron extracts were associated with significantly greater improvements in depressive symptoms. Additionally, these treatments demonstrated higher efficacy in participants with atypical depression compared to other patients, with response rates of 65% versus 35% [77].

A meta-analysis conducted in 2017 [88] included six clinical trials (lasting 4 to 8 weeks) involving 377 patients with depression, comparing either turmeric or curcumin to placebo. The results demonstrated a significant effect in favor of turmeric in reducing depressive symptoms. Another meta-analysis carried out in 2016 [68] concluded from subgroup analyses that curcumin exhibited the greatest antidepressant effects when administered to middle-aged adults, for longer treatment durations and at higher doses.

Two randomized controlled trials using 60–90 mg of concentrated *Crocus sativus* extract demonstrated a significant improvement in depression compared to placebo, measured by the Hamilton Depression Rating Scale (HAMD); in the first study [74], the extract was prepared from the stigmas of the flower, while in the second study [76], the extract was obtained from the petals. Equivalent effects on HAMD scores were observed in three randomized controlled trials comparing *Crocus sativus* stigma extract with imipramine and fluoxetine [89,90]. Clinical studies identified anxiety, tachycardia, nausea, dyspepsia, and appetite changes as possible side effects, although their statistical occurrence was not significant compared to placebo [74,76].

A double-blind randomized trial conducted over 12 weeks with 60 participants demonstrated that a daily dose of 50 mg of saffron stigma extract significantly improved mood and reduced anxiety compared to placebo [79].

As shown in Table 3, these clinical studies provide evidence supporting the therapeutic potential of several medicinal plants; however, variations in study design, dosage, and extract standardization indicate the need for further rigorous research.

### 2.4. Toxicological Risks Associated with the Use of Dietary Supplements

Over the past few decades, the global use of dietary supplements to manage anxiety, depression, and sleep disorders has grown substantially. These products are often perceived by consumers as safe and effective alternatives for supporting mental well-being [1]. Nevertheless, inappropriate or unsupervised use of these supplements may be associated with clinically relevant adverse reactions, including potentially serious outcomes [92].

This section examines the toxicological risks associated with the use of dietary supplements that affect the central nervous system, highlighting the importance of a cautious and informed approach to their consumption—particularly in the context of limited regulatory oversight and the lack of rigorous clinical evaluation of their safety and efficacy.

One of the most extensively documented toxicological concerns relates to kava kava, particularly its association with hepatotoxicity. Cases of hepatitis, cirrhosis, and acute liver failure have been reported, including severe cases progressing to liver transplantation, even after short-term or moderate use [93,94,95]. Proposed mechanisms include glutathione depletion, oxidative stress, and mitochondrial dysfunction. The risk appears to be higher with low-quality extracts or inappropriate plant parts. In addition to hepatic injury, kava use has been linked to neurological adverse effects such as sedation, ataxia, cognitive impairment, and, in rare cases, psychiatric disturbances, including suicidal behavior. Long-term, high-dose consumption may also result in dermatological manifestations, such as reversible kava dermopathy [96,97,98,99].

Vignier et al. [99] reported an association between high levels of kava consumption and suicidal behavior in adolescents. Although kava is primarily used for its anxiolytic effects, excessive or prolonged intake may result in central nervous system depression, emotional blunting, mood dysregulation and reduced dopaminergic and noradrenergic activity in limbic–prefrontal circuits involved in mood regulation. In vulnerable individuals, such effects may aggravate depressive symptoms and impair emotional control, thereby contributing indirectly to increasing susceptibility to suicidal ideation or behavior.

Another relevant example is *Passiflora incarnata,* which is generally considered well-tolerated; however, adverse effects including nausea, vomiting, excessive drowsiness, dizziness, and, rarely, cardiac arrhythmias (e.g., QT prolongation and ventricular tachycardia) have been reported [100,101,102]. Prolonged or high-dose use may contribute to daytime fatigue and, in susceptible individuals, impose additional hepatic strain [103,104].

High doses or prolonged use of *Valeriana oficinalis* may result in excessive sedation, dizziness, confusion, gastrointestinal symptoms, and, rarely, cardiovascular or respiratory complications. Long-term administration of valerian products may occasionally result in cardiac disturbances, headaches, mydriasis, restlessness, and insomnia [82].

Adverse reactions to *Hypericum perforatum* extract in the clinical treatment of depression include common effects such as skin redness and itching (erythema and pruritus), dizziness, constipation, fatigue, and anxiety, as well as less frequent but more severe events including photosensitivity, acute neuropathy, episodes of mania, and serotonin syndrome, particularly when administered concomitantly with other antidepressant medications [105,106].

Although generally considered safe at therapeutic doses, *Crocus sativus* L. has been associated with mild adverse effects in volunteer studies, including gastrointestinal discomfort, nervousness, dry mouth, and increased sweating. At higher doses (>1000 mg/day), saffron may cause vomiting, diarrhea, bleeding, hypotension, and, in rare cases, abnormal vaginal bleeding [107,108].

Finally, increasing evidence suggests that ashwagandha may be associated with hepatotoxicity. Reported cases describe cholestatic or mixed liver injury accompanied by jaundice, pruritus, and gastrointestinal symptoms, emphasizing the need for caution, especially in individuals with pre-existing liver disease [109,110].

As illustrated in Table 4, while herbal supplements are often perceived as safe, case reports reveal that serious toxicological effects—ranging from hepatotoxicity to neurological and dermatological reactions—can occur, emphasizing the need for careful monitoring and informed use.

### 2.5. Pharmacological Risks of Anxiolytic, Antidepressant, and Sedative Supplements

Pharmacologically active plant compounds, similar to conventional drugs, serve as substrates for metabolizing enzymes and their induction or inhibition can influence drug pharmacokinetics leading to clinically relevant interactions and contraindications for concomitant use [111,121,122]. Herb–drug interactions follow the same pharmacokinetic principles (alterations in plasma concentration of the drug) and pharmacodynamic mechanisms (drug interactions at the receptor level of target organs) as drug–drug interactions. Most psychotropic drugs are metabolized by cytochrome P450 isoenzymes, making them susceptible to pharmacokinetic interactions when co-administered with herbal preparations or dietary supplements [85,122,123,124,125].

St. John’s Wort (*Hypericum perforatum* L.) induces CYP3A4 and, to a lesser extent, CYP2C9 and CYP2C19 [126]. This enzyme induction may decrease plasma concentrations of benzodiazepines, antidepressants, and other psychotropic drugs, potentially causing withdrawal-like symptoms or reduced therapeutic efficacy. Concomitant use with SSRIs or SNRIs can also increase the risk of serotonin syndrome [127].

Several kavalactones in kava (e.g., methysticin, dihydromethysticin) inhibit multiple CYP450 isoenzymes (CYP1A2, 2C9, 2C19, 2D6, 3A4, 4A9/11), highlighting a high potential for pharmacokinetic interactions with other herbal or conventional medications metabolized via these pathways [128].

The active constituents of *Valeriana officinalis* and *Passiflora incarnata* may enhance the inhibitory activity of benzodiazepines that bind to GABA receptors, potentially leading to severe adverse effects [129].

The sedative effect exerted by *Passiflora incarnata* may potentiate the effects of medications with sedative properties, such as benzodiazepines (e.g., diazepam and lorazepam), barbiturates, or other drugs used in the treatment of anxiety. This interaction can enhance drowsiness, increase dizziness, and impair concentration [129]. When administered concomitantly with SSRIs or SNRIs, there is a risk of developing serotonin syndrome—a rare but severe condition caused by excessive serotonin levels in the brain. This may lead to symptoms such as agitation, tremor, hyperthermia, excessive sweating, and cardiac disturbances [130,131].

*Withania somnifera* (Ashwagandha) has been associated with the highest number of reported adverse events in the context of concurrent use with antidepressants and adaptogens, likely due to its widespread use as a dietary supplement [132]. Most interactions have been documented with selective serotonin reuptake inhibitors (SSRIs). Proposed mechanisms include: (1) pharmacokinetic interactions via cytochrome P450 isoenzymes involved in antidepressant metabolism, and (2) potentiation of adverse reactions due to overlapping pharmacodynamic profiles. Specifically, *Withania somnifera* extracts may inhibit CYP3A4 and CYP2D6 enzymes, significantly affecting the pharmacokinetics of antidepressants metabolized through these pathways [133]. Enzymatic inhibition may increase plasma concentrations of antidepressants, amplifying associated adverse reactions. For example, escitalopram co-administration has been linked to myalgia (Numeric Rating Scale > 5), epigastric pain, nausea, vomiting, restless legs syndrome, and severe nonproductive cough. With paroxetine, generalized myalgia (NRS 5) and ophthalmalgia were reported, while reboxetine was associated with testicular pain, ejaculatory dysfunction, and pain during ejaculation [134].

Table 5 summarizes reported interactions between conventional drugs and plant-derived substances, highlighting potential pharmacokinetic and pharmacodynamic effects, clinical manifestations, and management considerations.

### 2.6. Adulteration of Dietary Supplements

The adulteration of dietary supplements refers to the accidental contamination or deliberate introduction (also known as spiking) of active substances such as stimulants, anabolic agents, or pharmaceutical compounds, which are included on official lists of prohibited substances [138].

A common type of adulteration, particularly prevalent in the dietary supplement industry, involves replacing authentic ingredients with cheaper or ineffective substitutes, adding hazardous contaminants, or using inert substances intended to artificially increase volume. These practices significantly compromise the quality, safety, and efficacy of the product and mislead consumers [139,140,141]. Adulteration can also introduce toxic substances such as heavy metals, pesticides, or hazardous chemicals [141].

Adulterants introduced during the manufacturing process can trigger allergic reactions or sensitivities, particularly when authentic ingredients are replaced or contaminated. These reactions may include skin rashes, itching, swelling, or, in more severe cases, anaphylactic responses [140].

Regular consumption of such products may result in acute or chronic toxicity, manifested through symptoms like nausea, vomiting, and liver, kidney, or neurological damage [141]. Counterfeit products may contain irregular concentrations of active compounds, leading to imprecise dosing. This affects treatment efficacy, reducing therapeutic benefits or causing unexpected side effects due to product variability [142].

A particularly important concern is the chemical adulteration of products, which can pose serious risks to consumer health. To date, numerous reports have documented cases of herbal medicines and dietary supplements being adulterated while claiming to be completely natural. In reality, these products contained undeclared synthetic drugs intended to enhance their therapeutic effects [143].

According to data published in the FDA’s Health Fraud Product Database (2007–2023), a total of 1967 products were identified as fraudulent, of which 1264 (64.2%) contained undeclared pharmacologically active substances [144]. At the level of the European Union, it has been observed that many supplements are adulterated by the addition of various categories of active compounds. The most commonly detected were phosphodiesterase type 5 (PDE-5) inhibitors—37%—stimulants—34%—and anorectics and laxatives—14%. Other identified substances included cannabinoids (5%), nootropics (4%), anabolic androgenic steroids (2%), and various pharmaceuticals such as antibiotics and hypnotics (4%) [144].

Anxiolytic adulterants such as benzodiazepines (e.g., diazepam, clonazepam, alprazolam) have been listed among the types of illicit substances frequently added to dietary supplements, representing a common practice [145]. A frequently identified drug in adulteration cases is the antidepressant fluoxetine [145]. Beyond its efficacy in treating clinical depression, fluoxetine is also used in the treatment of eating disorders such as bulimia nervosa, as well as in obsessive–compulsive disorder [146].

Compared to the data provided by the FDA, the European Union has recorded a higher number of alerts concerning dietary supplements containing substances that affect brain function. Among these, two cases were identified in which supplements contained lithium—a drug used to stabilize mood in bipolar disorder and manic episodes—and vinpocetine, a medication commonly prescribed to improve cerebral circulation and to treat symptoms associated with cognitive disorders and ischemic stroke [147]. Previously, the RASFF reported the presence of α-methylphenylethylamine, a substance therapeutically used in the treatment of neurological disorders such as attention deficit hyperactivity disorder (ADHD) [17].

In November 2023, the FDA issued an official warning to consumers regarding the risks associated with products containing tianeptine, a pharmaceutical compound not approved in the United States, falsely promoted for enhancing brain function and alleviating symptoms of anxiety and depression. The use of these products has been linked to serious adverse events, including seizures and loss of consciousness, which have required hospitalization in multiple cases [148].

A commonly found ingredient in dietary supplements aimed at neurocognitive health is 5-hydroxytryptophan (5-HTP), a naturally derived substance extracted from the seeds of the *Griffonia simplicifolia* plant. In the body, 5-HTP is endogenously produced through the conversion of the essential amino acid tryptophan and serves as a direct precursor of serotonin—a neurotransmitter involved in the regulation of mood, reward, and cognitive functions. Additionally, 5-HTP can be further converted into melatonin, a key hormone in regulating the circadian rhythm and the sleep–wake cycle [149].

Gamma-aminobutyric acid (GABA) is another compound frequently used in the United States as an active ingredient in dietary supplements aimed at reducing anxiety and improving mood. Although its marketing is permitted both in the US and in several member states of the European Union, GABA has been subject to two notifications within food safety systems. One of these concerned the use of unauthorized health claims, while the other related to the classification of GABA as a medicinal substance by the Finnish Medicines Agency, which entails stricter regulations regarding its distribution and use in that country [150].

In 2019, public notifications were issued regarding two sleep-enhancing supplements found to contain sedative-hypnotics such as eszopiclone and zopiclone [151]. During the same period, two other substances with central nervous system activity—huperzine A and dimethylaminoethanol (DMAE)—were reported in the RASFF due to their presence in dietary supplements [151].

Huperzine A, an alkaloid isolated from *Huperzia serrata*, a species traditionally employed in Chinese medicine, has been shown to exert pronounced anticholinesterase activity in pharmacological studies. Currently, huperzine A is widely incorporated into dietary supplements commercialized in the United States, primarily with claims of supporting cognitive function, enhancing memory performance, and improving attentional capacity. By contrast, within the European Union, huperzine A is regarded as an unauthorized constituent of dietary supplements, irrespective of its source, and was cited in 27 official notifications between 2020 and 2023 [152].

DMAE, a choline derivative with effects on the cholinergic system, is recognized as an ingredient in dietary supplements sold in the United States due to its natural presence in fish such as salmon, mackerel, and sardines. However, DMAE is considered an unauthorized substance in Europe. This compound has been used in pharmacotherapy in both regions to treat central nervous system disorders, including dementia associated with cholinergic neuron dysfunction in elderly individuals, behavioral problems, ADHD, and for improving memory, concentration, and learning processes. In the European Union, DMAE has been responsible for six notifications in the RASFF over the past four years [17].

## 3. Materials and Methods

A literature search was performed using major international scientific databases—PubMed, Scopus, and Web of Science—in order to identify relevant publications addressing the pharmacological and toxicological risks associated with dietary supplements acting on the central nervous system, particularly those with anxiolytic, antidepressant, or sedative properties. The present study was designed as a narrative review.

The search strategy combined free-text keywords and controlled vocabulary terms related to plant-based supplements and their potential pharmacological and toxicological risks. Search terms included combinations of “dietary supplements”, “anxiolytic”, “antidepressant”, “sedative”, and terms referring to safety outcomes such as “toxicity”, “adverse effects”, “drug interactions”, “pharmacological safety”, and “side effects”. These terms were combined using Boolean operators (AND/OR) to identify studies addressing supplements with central nervous system activity and associated risks. In addition, specific plant-derived compounds and products (e.g., valerian, St. John’s wort, melatonin, GABA, 5-HTP) were included to ensure comprehensive coverage of relevant clinical and toxicological evidence.

This review primarily focused on research published over the past two decades, reflecting the growing scientific interest in dietary supplements with central nervous system activity, while also including selected earlier key publications to provide background and foundational insights.

The final selection was restricted to peer-reviewed journal articles presenting original research or comprehensive reviews relevant to the evaluation of pharmacological and/or toxicological risks associated with supplements that influence mental state.

### 3.1. Study Selection

Study selection was conducted using predefined inclusion and exclusion criteria. Eligible publications included clinical studies, observational studies, case reports, and relevant toxicological reports addressing plant-based dietary supplements with central nervous system activity and associated safety concerns (e.g., adverse effects, toxicity, or herb–drug interactions).

Studies not related to dietary supplements affecting anxiety, depression, or sleep disorders, publications lacking original clinical or toxicological data, and non-scientific sources were excluded. Titles and abstracts were screened for relevance, followed by full-text evaluation of potentially eligible articles.

### 3.2. Inclusion Criteria

- Investigated plant-based dietary supplements with central nervous system activity, particularly those with anxiolytic, antidepressant, or sedative effects;
- Reported data on safety, toxicity, adverse effects, or herb–drug interactions;
- Included clinical studies, observational studies, case reports, toxicological investigations, or relevant systematic reviews;
- Published in peer-reviewed scientific journals;
- Written in English.

### 3.3. Exclusion Criteria

- Studies not addressing supplements with central nervous system effects;
- Articles focusing exclusively on therapeutic or nutritional benefits without safety or risk data
- Editorials, commentaries, letters, or publications without scientific or clinical data;
- Studies lacking sufficient methodological quality or relevant safety information.

A total of 300 records were initially identified through database searches (280 records) and additional sources (20 records). After removing duplicates, 280 unique records remained for screening.

These records were screened by title and abstract, resulting in the exclusion of the records that did not meet the inclusion criteria. The full texts of the remaining 156 articles were assessed for eligibility.

## 4. Conclusions

Dietary supplements with anxiolytic, antidepressant, and sedative properties may represent useful complementary options for mild-to-moderate symptoms, particularly when high-quality, standardized extracts such as *Hypericum perforatum*, *Valeriana officinalis*, *Passiflora incarnata*, *Withania somnifera*, *Piper methysticum* and *Crocus sativus* are used. However, despite widespread perceptions as inherently safe, these products present significant pharmacological and toxicological risks, including clinically significant herb–drug interactions, excessive sedation, serotonin syndrome, and adverse effects associated with adulterated or undeclared compounds.

Responsible use requires thorough risk–benefit evaluation, clinical monitoring, and informed patient counseling. Strengthened post-market surveillance, improved labeling transparency and enforcing consistent quality standards remain essential to enhance consumer safety and therapeutic reliability.

Current evidence is limited by heterogeneous study designs, variable dosages, and insufficient long-term safety data. Standardized clinical trials, mechanistic studies, and strengthened pharmacovigilance initiatives are needed to better define efficacy, optimal dosing, and comprehensive safety profiles, thereby supporting a more rational and evidence-based use of plant-derived psychotropic supplements.

## Figures and Tables

**Table 1:** Comparative analysis of European regulations on food supplements and drugs.

Criterion	Food Supplements	Medicines
Legal Definition	Foods intended to supplement the diet with nutrients or other substances with nutritional effects [6]	Substances or combinations intended to treat, alleviate, or prevent diseases [7]
Main Regulation	Directive 2002/46/EC, Regulation 1924/2006 [6,8]	Directive 2001/83/EC and Regulation (EC) No 726/2004 [7]
Pre-Market Authorization	No prior authorization required (notification to national authority) [6]	Mandatory—Marketing Authorization (MAA) after rigorous scientific evaluation [7]
Efficacy Evaluation	Not required. No need to prove it “works” [6]	Mandatory. Efficacy must be demonstrated through controlled clinical trials [7]
Safety Evaluation	Only at the ingredient level (sometimes incomplete); the final product is not tested [9]	Comprehensive. Includes preclinical testing and human clinical trials [10]
Quality Control	Limited, mainly post-market [6]	Rigorous—GMP (Good Manufacturing Practice) standards, batch-by-batch testing [10]
Health Claims	Only those approved by EFSA (European Food Safety Authority). Most claims for herbal products are not allowed [9]	Allowed if based on clinical data—can make explicit claims about disease treatment [11]
Advertising	Cannot suggest treatment, prevention, or cure of diseases [6]	Strictly regulated—must reflect the authorized indications of the medicine [7]
Online Marketing	Widespread, often with weak oversight [6]	Possible, but strictly regulated and monitored [7]
Post-Market Monitoring	Limited, occasional inspections by national authorities [6]	Mandatory—pharmacovigilance, periodic safety reports [7]
Adverse Effects	Not systematically monitored [6]	Mandatory reporting—by both professionals and patients [7]
Ingredient Approval	Only EU-harmonized vitamins and minerals. Others vary by country [6]	Active ingredients approved by EMA (European Medicines Agency) or national authorities, based on evidence [10]

**Table 2:** Examples of plants with anxiolytic, antidepressant, and sedative effects: mechanisms of action and associated adverse reactions.

Plant Name	Active Compounds of Interest	Mechanism of Action	Uses	Adverse Reactions	References
St. John’s Wort (*Hypericum* *perforatum*)	Hyperforin, Hypericin	Non-specific inhibition of serotonin, norepinephrine, and dopamine reuptake, leading to increased levels of these neurotransmitters in synapses	Depression, anxiety, agitation	Sedation, fatigue, headache, insomnia, restlessness, seizures, confusion, delirium, hypertension, tachycardia, nausea	[45]
Passionflower (*Passiflora incarnata*)	Flavonoids (Apigenin, Luteolin, Quercetin, Kaempferol) glycosides (vitexin, isovitexin, orientin, and isoorientin)	Increases brain GABA levels, exerting inhibitory effects on neuronal excitability	Anxiolytic, antidepressant, relaxant, sedative, sleep-promoting	Confusion, dizziness, sedation, ataxia, nausea, vomiting, drowsiness, tachycardia	[52]
Valerian (*Valeriana officinalis*)	Volatile oil, valerenic acid	Modulates GABA receptors, adenosine, dopamine, norepinephrine; reduces cortisol	Tranquilizer-sedative, sleep-promoting	Sedation, dizziness, excitability, headache, diarrhea, hepatotoxicity, tachycardia	[57,58,59]
Kava (*Piper methysticum*)	Kavalactones: kavain, dihydrokavain, methysticin, dihydromethysticin, yangonin	Reversible inhibition of MAO-B enzyme, increases GABA_A_ receptor density, blocks voltage-gated Na^+^-channels	Low doses: stimulant, relaxant, anxiolytic; high doses: sedative, sleep-promoting	Drowsiness, agitation, confusion, delirium, psychosis, hallucinations, seizures, hyperthermia, coma	[60,63]
Ashwaganda (*Withania somnifera*)	Withanolides	Regulates hormonal levels, decreases cortisol, normalizes adrenal gland function	Adaptogen, sedative, stimulant, anti-inflammatory, neuroprotective, cardioprotective	Diarrhea, nausea, arrhythmias, hypotension, breathing difficulties	[72]
Saffron (*Crocus sativus*)	Crocin, picrocrocin, volatile compounds including safranal	Influences serotonin, dopamine levels, and GABAergic system	Anxiolytic, antidepressant, neuroprotective	Rash, nausea, vomiting, abdominal pain, hypotension	[65,66]
Turmeric (*Curcuma longa*)	Curcumin	Modulates neurotransmitter concentrations (serotonin, dopamine, norepinephrine), reduces inflammatory factors (TNF-α, IL-1β, COX-2)	Anxiolytic, antidepressant, neuroprotective, antioxidant, anti-inflammatory	Gastrointestinal disturbances (bloating, nausea, diarrhea), rash, liver function impairment	[67,68,69]

**Table 3:** Clinical studies on the effects of plant extracts in anxiety, depression, and insomnia disorders.

Disorder Type	Study/Population	Intervention/ Active Control/ Treatment Duration	Results	Reference
Depression	Randomized, double-blind, 3 groups (135 patients)	900 mg/day standardized St. John’s Wort extract Active control: 20 mg/day fluoxetine 12 weeks	HAMD scores were lower in the St. John’s Wort group compared to fluoxetine, indicating a greater improvement in depressive symptoms. Remission rates were higher with St. John’s Wort.	[75]
Moderate Depression	Multicenter, double-blind, 388 patients	Hypericum STW3-VI extract 900 mg/day Active control: 20 mg citalopram 6 weeks	Hypericum extract demonstrated better safety and tolerability compared to citalopram and is a good alternative antidepressant for outpatient treatment.	[78]
Preoperative Anxiety	Double-blind, 60 ambulatory surgery patients	Passiflora tablets 500 mg Active control: Oxazepam 10 mg	Both reduced preoperative anxiety; effects on postoperative psychomotor function were similar.	[54]
Generalized Anxiety Disorder	Randomized, double-blind, 36 patients	Passiflora extract 45 drops/day Active control: Oxazepam 30 mg/day 28 days	Both treatments produced a similar reduction in Hamilton Anxiety scores, demonstrating comparable efficacy.	[55]
Opioid Dependence	Randomized, double-blind, 65 patients	Passiflora extract 60 drops/day + Clonidine 0.8 mg/day Control: Clonidine 0.8 mg/day, 14 days	Combination therapy was more effective than clonidine alone in managing withdrawal symptoms.	[91]
Chronic Insomnia	Randomized, double-blind, ages 18–73	Standardized valerian root extract 600 mg/day Active control: Oxazepam 10 mg 6 weeks	Valerian improved sleep quality comparably to oxazepam but with fewer side effects and no dependency risk.	[82]
Preoperative Anxiety	Double-blind clinical trial, 60 patients (15–60 years)	10 drops valerian oil Active control: Diazepam 5 mg in 50 mL water 30 days	Valerian was nearly as effective as diazepam in reducing anxiety symptoms with fewer adverse effects reported.	[83]
Major Depressive Disorder	Open-label, randomized, 80 patients	Ashwagandha 250 mg twice daily Active control: Sertraline 3 months	Ashwagandha significantly reduced anxiety and depressive symptoms, showing comparable efficacy to sertraline without significant side effects.	[84]
Cognitively Healthy Adults	Randomized, double-blind, 130 adults	300 mg Ashwagandha root extract (Prolanza™), sustained-release capsule once daily Placebo 90 days	Ashwagandha improved memory, attention, sleep quality, and psychological well-being; stress levels decreased. Treatment was safe and well tolerated.	[85]
Generalized Anxiety Disorder	Multicenter clinical trial, 129 patients	400 mg/day Kava LI 150 standardized to 30% kavapyrone Buspirone 10 mg/day Opipramol 100 mg/day 8 weeks	Kava showed equal efficacy to reference treatments; 75% of patients had ≥50% reduction in Hamilton Anxiety scores and ~60% achieved full remission; well tolerated.	[86]
Generalized Anxiety Disorder	Randomized, double-blind, 37 patients	Kava (standardized to 70 mg kavalactones) Initial dose 140 mg/day, increased to 280 mg/day Placebo 4 weeks	Improvements seen in both groups, with no significant difference compared to placebo.	[87]
Depression	Randomized, double-blind, 123 adults	(1) 250 mg curcumin (BCM-95^®^) twice daily (2) 500 mg curcumin twice daily (3) 250 mg curcumin + 15 mg saffron twice daily (4) Placebo 12 weeks	Curcumin and saffron treatments showed significantly greater improvement in depressive symptoms compared to placebo.	[77]

**Table 4:** Adverse effects of herbal supplements—case reports.

Herbal Supplement	Case/Description	Clinical Manifestations	Complications	Treatment/ Evolution	Reference
Kava kava	Female, 14 y.o., liver failure after 4 months of use; required liver transplant	Jaundice, severe liver failure	Hepatic necrosis, severe failure	No response to treatment, liver transplant	[111]
Kava kava	11 cases (Germany, USA, Switzerland) of acute liver failure	Acute liver injury, jaundice	Severe liver failure	Surgery; some cases required transplant	[111,112,113,114,115,116,117,118]
Valerian + GABA	Female, ~48 y.o., bipolar disorder with catatonia; encephalopathy	Encephalopathic delirium, altered consciousness	Severe encephalopathy	Clinical clarification after detailed anamnesis	[117]
*Passiflora incarnata*	Female, 34 y.o., therapeutic doses; nausea, vomiting, drowsiness, long QT	Nausea, vomiting, excessive sleepiness, ventricular arrhythmia	Severe cardiac risk	ECG monitoring, IV hydration; good outcome	[118]
Saffron (*Crocus sativus*)	Female, 35 y.o., excessive ingestion for anxiety	Nausea, vomiting, dizziness, tachycardia	Ventricular tachycardia	IV hydration, monitoring; full recovery	[119]
St. John’s Wort *Hypericum perforatum*	Female, 61 y.o., 3 years of oral use	Pruritic erythematous rashes	Cutaneous photosensitivity	Discontinuation of supplement; full recovery	[120]

**Table 5:** Interactions of drugs with plant substances.

Drug	Herb	Result of Interaction	References
Escitalopram	*Withania somnifera*	Myalgia, epigastric pain, nausea, vomiting, restless legs syndrome, dry cough	[134]
Fluoxetine	Kava	Excessive sedation	[134]
Duloxetine	St. John’s Wort	Withdrawal syndrome, anxiety worsening	[134]
Haloperidol	St. John’s Wort	Anxiety, worsening of qualitative consciousness disorders	[135]
Quetiapine	St. John’s Wort	Insomnia, anxiety worsening	[92]
Diazepam	Kava	Excessive sedation	[92,136]
Clonazepam	St. John’s Wort	Withdrawal syndrome, anxiety worsening, insomnia, tremor, sinus tachycardia	[137]

## Data Availability

No new data were created or analyzed in this study. Data sharing is not applicable to this article.
